# Dietary counselling plus omega-3 supplementation in the treatment of generalized anxiety disorder: protocol for a randomized wait-list controlled pilot trial (the “EASe-GAD Trial”)

**DOI:** 10.1186/s40814-023-01414-y

**Published:** 2023-11-10

**Authors:** Monique Aucoin, Laura LaChance, Inge van der Wurff, Sean Miller, Umadevi Naidoo, Andrew Jenkins, Kieran Cooley

**Affiliations:** 1 Canadian College of Naturopathic Medicine, Toronto, Canada; 2 University of Guelph, Guelph, Canada; 3 McGill University, Montreal, Canada; 4 St. Mary’s Hospital, Montreal, Canada; 5 Health Psychology, Faculty of Psychology, Open University of the Netherlands, Utrecht, Netherlands; 6 Peer Connections Manitoba, Winnipeg, Canada; 7 Massachusetts General Hospital, Boston, USA; 8 Harvard Medical School, Boston, USA; 9 Lipid Analytical Laboratory, Guelph, Canada; 10 Southern Cross University, Lismore, Australia; 11 University of Technology Sydney, Ultimo, Australia; 12 Pacific College of Health and Science, San Diego, USA

**Keywords:** Nutrition, Diet, Anxiety, Psychiatry, Mental health, Lifestyle, Omega-3

## Abstract

**Background:**

Anxiety disorders are prevalent and disabling conditions involving excessive worry and tension. Generalized anxiety disorder (GAD), the most common anxiety disorder, affects 5% of individuals from high-income countries and many individuals report that treatment options are not accessible, effective, or tolerable. Clinical evidence suggests that nutrition interventions, based on the Mediterranean diet and supplementation of omega-3 fatty acids, can significantly improve symptoms of depression; however, the effect of nutrition interventions on anxiety symptoms has not been studied in a clinical population. The primary objective of the present study is to assess the feasibility and acceptability of a dietary counselling and omega-3 fatty acid supplementation intervention delivered to adult women with GAD. The secondary objectives include assessing changes in anxiety symptom severity, assessing changes in quality of life, assessing changes in biomarkers, and evaluating the components of the program.

**Methods:**

This study is a randomized, wait-list controlled pilot trial delivering a 12-week, dietary counselling intervention and omega-3 supplementation to 50 adult women with GAD. Participants will complete seven individual counselling sessions which include education, personalized recommendations, mindful eating techniques, motivational interviewing, and goal setting. They will be provided with recipes, instructional videos, and food items. The intervention is designed based on the Social Cognitive Theory and previous research that has been done by the author team to identify dietary constituents with the most evidence to support their use in the treatment of anxiety disorders. Questionnaires and blood work will be completed at baseline, after the waiting period (for those in the waitlist group), and after the intervention.

**Discussion:**

Results from this study will lay the foundation for future large-scale studies in this area and may provide preliminary evidence of the role of diet counselling and omega-3 supplementation in the management of GAD. Research on the role of nutrition in psychiatric care has been identified as a priority by a number of international organizations. The present trial directly addresses the call for the research that is most needed to advance the field.

**Trial registration:**

This protocol was registered at ClinicalTrials.gov on October 10, 2022; NCT05573672.

Trial sponsor: The Canadian College of Naturopathic Medicine, 1255 Sheppard Ave E, Toronto, ON M2K 1E2, 416–498-1255.

Steering committee: Composed of MA, LL, KC, IvdW, SM, UN, AJ. The committee meets monthly to oversee the trial.

Protocol identifier: CCNM_EASe-GADCT_2201v4.

**Supplementary Information:**

The online version contains supplementary material available at 10.1186/s40814-023-01414-y.

## Background

Generalized anxiety disorder (GAD) is a common psychiatric disorder involving high levels of worry or tension. The symptoms are persistent and distressing and they interfere with an individual’s functioning [1]. Cross-national data suggest that 5% of adults from high-income countries are affected by symptoms compatible with GAD in their lifetime [2], and that the illness exerts a large negative impact on quality of life [3]. Available treatment options include pharmacotherapy and psychotherapy; however, due to adverse effects, high cost, and incomplete resolution of symptoms, many patients report that these options are inaccessible, ineffective, or intolerable [4] and the percentage of patients adequately treated is estimated to be as low as 20 to 32% [5]. The result is a large personal and social cost and a need for alternative or adjunctive therapeutic options.

There is growing evidence that nutrition is an important modifiable risk factor for many illnesses, including psychiatric illnesses. While the field of nutritional psychiatry research is rapidly expanding, research on anxiety disorders lags behind research related to depression. Observational evidence shows an association between poor diet quality and high prevalence/severity of anxiety disorders [6–8]. Recently, several randomized, controlled trials involving participants with depressive disorders have reported significant improvement in depression symptoms following dietary interventions aimed at improving diet quality and increasing consistency with the Mediterranean diet [9–12] alone or in combination with omega-3 fatty acid supplementation [13]. A recent systematic review identified 17 studies that assessed the effect of diet modification on depression and anxiety symptoms [14]. Of these, ten assessed anxiety outcomes; however, only one of these studies enrolled participants with clinically diagnosed anxiety disorders [10], the remaining assessed changes in anxiety symptoms in patients with physical illnesses such as cardiometabolic illness or healthy participants. In this single study [10], participants were included if they met the criteria for a major depressive episode; however, the mean anxiety severity rating score was consistent with the presence of clinically significant anxiety. Following a 12-week diet counselling intervention, the mean anxiety score decreased significantly compared with the control intervention [10]. The low or absent baseline levels of anxiety symptoms in the remaining studies may have precluded the detection of a change. Additionally, findings from studies involving non-psychiatric populations have unclear applicability to psychiatry populations. Overall, the effect of diet modification on anxiety symptoms in clinical populations is unknown. There have been multiple calls for clinical research that modifies the dietary patterns of individuals with anxiety disorders [14, 15].

The Mediterranean Diet is the pattern of food traditionally consumed by individuals from the Mediterranean region. It includes a high intake of vegetables, fruit, whole grains, legumes, olive oil, seafood, and water, a moderate intake of poultry, cheese, and yoghurt, and a low intake of meats and sweetened foods. The Mediterranean diet has been extensively researched and higher levels of consistency with this diet pattern have been associated with a large number of positive health outcomes including decreased risk of cancer [16], cardiovascular disease [17], and all-cause mortality [18]. Early clinical research using diet interventions in the treatment of depression has used a version of the Mediterranean diet [10, 12, 13]. Omega-3 fatty acids are important dietary factors related to mental health. A recent meta-analysis found an association between omega-3 supplementation and a reduction in anxiety symptom scores in clinical trials [19].

The relationship between diet and mental health is complex and bidirectional. While prospective trials assessing baseline diet and mental health outcomes at follow-up suggest that poor diet quality may play a role in the development of mental illness [6], there is also evidence suggesting that the presence of psychiatric symptoms may influence diet choices. Animal studies have reported that a highly palatable diet decreases behavioural and neuroendocrine markers of anxiety [20] and human data also suggest a connection between anxiety symptoms and “emotional eating” or “comfort eating”, defined as the consumption of food in response to negative affect in an effort to mitigate emotional symptoms [21]. This typically includes an increase in the amount of food and an increase in food with higher levels of calories, sugar, and fat [21, 22]. This pattern of eating behaviour contributes negatively to the diet quality of individuals with mental illness [23]. Individuals affected by anxiety experience more sweet cravings [24] and observational studies show that increased eating as a response to difficult emotions may decrease levels of perceived stress [25]. The results of numerous studies suggest that sex and/or gender may play a role in comfort eating and that women may be more susceptible to this phenomenon [23, 26, 27]. One large observational study found that women were nearly three times as likely as men to eat something as a coping strategy in response to emotional stress [28]. Human and animal studies have found an effect of oestrogen and progesterone cycles on comfort eating behaviours [20, 29]. Factors related to gender, such as eating restraint and disinhibition [23, 26], may also mediate this relationship. One approach that has been shown to improve emotional eating behaviour is called “mindful eating”, defined as “an approach to food that focuses on individuals’ sensual awareness of the food and their experience of the food” [30]. Participants in pilot trials using mindful eating interventions have reported improved eating behaviours [31].

Observational evidence suggests that food consumption and diet choices impact mental well-being. Preliminary clinical evidence suggests that diet modification intervention and omega-3 supplementation can improve symptoms of depression. The impact of these interventions on anxiety symptoms, among individuals with GAD, has not been assessed to date in a clinical trial. This protocol is for the *Eating and Supplementation for Generalized Anxiety Disorder study* (“EASe-GAD”), a randomized, wait-list controlled, pilot trial aimed at assessing the feasibility and acceptability of dietary counselling and omega-3 supplementation intervention delivered to women with GAD. This protocol was prepared using the SPIRIT checklist (Supplemental file 1).

## Methods

### Objectives

The primary objective of the proposed pilot clinical trial is to assess the feasibility and acceptability of delivering diet counselling plus supplementation intervention to adult women with generalized anxiety disorder (GAD) as measured by recruitment, retention, and intervention uptake. The secondary objectives are to evaluate changes in anxiety symptom severity, quality of life, diet quality, mindful eating behaviours, self-efficacy, and biomarkers, over the intervention period as compared to the waitlist control period. This trial will lay critical groundwork for a larger, adequately powered clinical trial, designed to assess the effectiveness of dietary counselling in the management of GAD.

### Trial design and setting

**Fig. 1:**
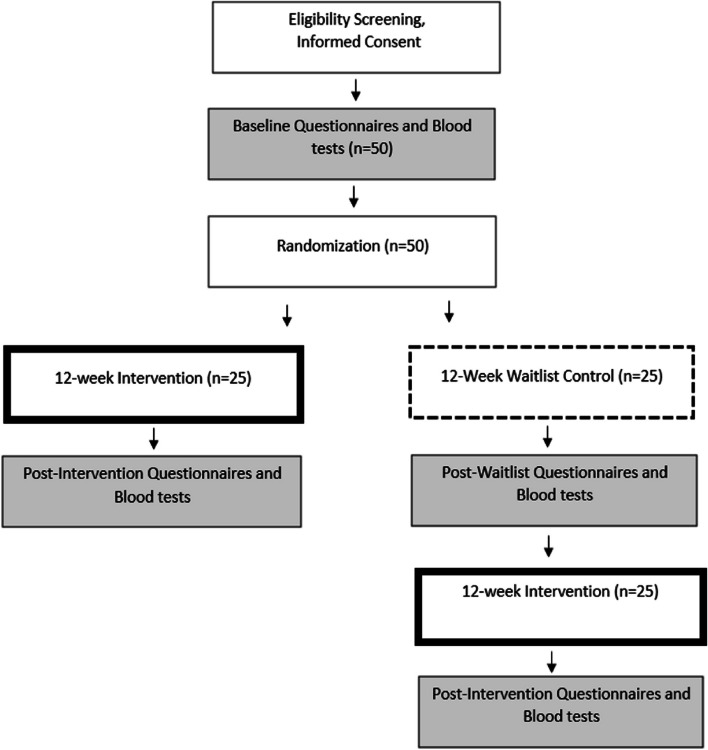
Diagram of participant flow through the trial

This pilot intervention study will recruit 50 adult women with moderate to severe GAD and provide a dietary counselling intervention in combination with an omega-3 supplement that also contains vitamin D. A randomized, waitlist-controlled design will be used (Fig. 1). The waitlist-control design was selected to allow for random allocation to a non-treatment condition while still allowing all participants to complete the intervention. Waitlist-control designs have been suggested to improve rigour and preserve ethical considerations for studies where participant blinding is difficult [32]. Twenty-five participants will be randomized to begin the intervention immediately and 25 participants will be randomized to observe a 12-week waitlist period before completing the intervention. This trial will take place at a single site. The study setting is the community clinic at the Canadian College of Naturopathic Medicine in Toronto, Canada. The study has been reviewed by the Research Ethics Board of the Canadian College of Naturopathic Medicine (CCNMREB040.Aucoin). Any amendments to this protocol will be communicated to the Research Ethics Board and Health Canada (the authority responsible for approval, conduct, oversight, and inspection of clinical trials in Canada) and modified on the trial registry. The design of this study involved participation from people with expertise in nutrition, psychiatry, research methods, laboratory testing, culinary education, behaviour changes, and lived experience with mental illness.

### Participants eligibility

#### Inclusion criteria

1. A person aged 18–65 years who currently identifies as a woman.
2. Primary diagnosis of generalized anxiety disorder based on a clinical interview with a psychiatrist using the Diagnostic and Statistical Manual of Mental Disorders (DSM-5) criteria.
3. Moderate to severe anxiety is defined as a Beck Anxiety Inventory score of 22 or higher.
4. Low quality diet is defined as a score of 8.5 or less on the MEDI-LITE tool.
5. All psychiatric medication, psychotherapy, and natural health products stable for the past 4 weeks.
6. Established therapeutic relationship with a family doctor, psychiatrist, or clinical psychologist as their primary source of mental health care.
7. Ability to swallow capsules.
8. Ability to read in English and provide informed consent.

#### Exclusion criteria

1. Currently meets DSM-5 criteria for obsessive–compulsive disorder, bipolar disorder, a psychotic disorder, an eating disorder, or substance use disorder based on clinical interview with a psychiatrist.
2. Current high level of suicidality was assessed by a trial psychiatrist using the Columbia Suicide Severity Scale.
3. Starting or changing the dose of a psychiatric medication, psychotherapy, or natural health products in the past 4 weeks.
4. Severe food allergies, intolerances, or aversions that would prevent the individual from modifying their diet (includes aversion to bovine gelatin which is a component of the fish oil supplement).
5. Current participation in a program, research study, or treatment plan involving diet or lifestyle modification.
6. Baseline Omega Score of > 5% (suggesting adequacy of omega-3 fatty acids).
7. Supplementation of greater than 4000 IU of vitamin D daily for at least 1 month in the previous 6 months.
8. Allergy to fish or any of the non-medicinal ingredients in the study product.

Participants who are assigned to the waitlist control, and who do not meet one or more of the criteria at the time of starting the intervention will still be permitted to participate. This data will be included in the analysis. If a participant begins a new medication or psychotherapy, or adjusts the dosage, during the waitlist period, this new treatment must be stable for 4 weeks prior to beginning the treatment phase of the study, even if this requires extending the waitlist period by up to 4 weeks.

The inclusion and exclusion criteria were designed with the intention of recruiting a sample of participants who are likely to be good candidates for the proposed intervention. Assessment of baseline diet quality, and inclusion of individuals with low quality at baseline, was completed in a similar trial [10]; similarly, trials utilizing omega-3 supplementation have highlighted the importance of including participants with sub-optimal baseline levels [33]. The participants are also similar with respect to non-modifiable factors that might influence the impact of the intervention. For example, there is evidence that emotional eating behaviours, which many influence the eating habits of people experiencing anxiety and depression, may be influenced by sex and/or gender and that women may be more affected [23]. In the absence of clear evidence differentiating the respective roles of sex and gender, we have elected to include participants who presently identify as women. We recognize that this may introduce greater heterogeneity within the sample with respect to hormonal status; however, we acknowledge that the sample of adult women will include variation in hormonal status related to hormonal birth control, menstrual and hormonal cycle irregularities and disorders, and medically induced or natural menopause. We have included a question in the demographic questionnaire about sex assigned at birth. This will be used to characterize the study participants. If a significant number of participants respond with an answer other than female, sub-analysis may be undertaken. Future studies can be undertaken to assess the impact of diet on men or people of other genders who experience anxiety disorders.

Participants with co-morbid depression will be eligible to participate in the intervention because of the high level of comorbidity with GAD [34]. The criterion related to recent high-dose vitamin D supplementation is included to identify potential participants who would exceed the maximum recommended daily allowance of vitamin D if they began taking the study product. Initially, this exclusion criterion was “Supplementation of greater than 1700 IU of vitamin D daily […]”; however, early results from screening identified a high number of interested individuals who were ineligible because of this criterion, and an amendment was made to make the study more inclusive without compromising on participant safety. In order to ensure that participants have access to adequate care, should they experience a serious worsening of their psychiatric illness during the study period, an established therapeutic relationship with a mental health professional is required.

### Sample size and recruitment plan

Because of the pilot nature of the present trial, a formal sample size calculation was not completed. No intervention study has attempted to deliver a dietary intervention to participants with GAD and thus, the expected effect is unclear. The results of this pilot trial will inform the sample size calculation for a full-scale trial.

Participants will be recruited through multiple strategies. Letters will be mailed to local mental health service providers including psychiatrists, counsellors, psychotherapists, and psychologists. The clinicians will be provided with a poster to display in their office and fliers to distribute directly to clients. Additional recruitment strategies will include online advertising through Facebook and other social media sites. This will include posts in existing, local anxiety/mental health groups and paid advertisements. If needed, newspaper advertisements may be used.

### Pre-study screening, randomization, and baseline evaluation

Interested participants who contact the study will complete a virtual screening visit. A member of the research team will administer the MEDI-LITE Score, and the Beck Anxiety Inventory and review the additional inclusion and exclusion criteria by phone or secure video call. If the participant meets these criteria, they will complete the clinical interview and Columbia Suicide Severity Scale with the trial psychiatrist via secure video platform. Following the completion of a verbal consent process, the participant will be mailed a kit for collecting a blood spot sample at home and will return the sample by mail for analysis of baseline omega-3 status.

Individuals who meet all study inclusion criteria will be contacted and invited to enrol in the study. There is no predetermined time between screening and baseline; as soon as all screening requirements are complete, enrollment and baseline data collection can begin. The baseline activities and data collection will take place through a combination of virtual and in-person activities. In an enrollment video call with the study coordinator, participants will complete the informed consent process by reviewing the informed consent form and asking questions. Following enrollment, baseline data collection will involve the completion of several questionnaires through a secure online platform (Supplemental file 2). If the participant does not have access to an internet-enabled device, they will be mailed a paper copy of the baseline questionnaires. The baseline assessment will also include the provision of a fasting blood sample. Following baseline data collection, participants will attend an in-person study visit. At this time, baseline height and weight will be measured, and the participants will be randomized. Randomization will be completed by the trial coordinator using a computer-generated internet-based randomization service provider using random permuted blocks. Following the assignment, the trial coordinator, intervention deliverer, and participants will be aware of allocation. Outcome assessors will be blind to allocation when measuring height and weight. Measurement of the omega-3 samples and interpretation of the data will be completed by a research team member who is blind to allocation. Data analysts will be blind to allocation; they will be provided with data from two groups of participants labelled groups 1 and 2.

For participants randomized to the waitlist control group, the baseline visit will conclude with instructions for the waitlist period. Participants randomized to the immediate start group will immediately begin the first dietary counselling session. There is no predetermined time between baseline data collection and commencement of the intervention; once all baseline electronic data are collected, participants may schedule the first in-person visit which will include the collection of the remaining baseline data and the first intervention session.

### The intervention

The intervention will consist of seven dietary counselling sessions delivered over 12 weeks (one session every 2 weeks). Three visits (the first, fourth, and seventh) will take place in person and the remaining will take place by secure video platform. If participants do not have access to a computer or the Internet, they may complete all sessions in person. In the event that COVID-19 restrictions change during study delivery, the number of virtual sessions may be altered in order to comply with local restrictions. The sessions will be 45 to 60 min in length.

### Intervention development

The dietary recommendations are based on the Mediterranean diet [35] with minor modifications based on the results of a recent scoping review which identified and collated all of the existing research on diet factors and anxiety [36]. The Mediterranean diet has been studied extensively; a meta-analysis has demonstrated an association between greater adherence to the Mediterranean diet and reduced risk of depression [37] and this diet has served as the intervention in other clinical trials where diet was used in the treatment of depression [10, 12, 13], with positive outcomes. The scoping review additionally suggested that the following dietary components were important for people with GAD: limitation of caffeine intake, the inclusion of culinary herbs and spices, green tea and herbal teas, dietary factors that support microbiome health, and an emphasis on adequate protein and lower glycemic index foods [36]. The Canadian Food Guide (2019) was utilized as a reference for national dietary recommendations and culturally relevant foods [38]. Because we expected the study participants to reflect the multicultural nature of Canada, we intentionally included foods and recipes reflecting a range of cultural backgrounds. Lastly, the dietary interventions and resources used in nutritional psychiatry studies that have previously reported benefits were reviewed in order to guide the design of the present intervention [39].

### Use of theory

The behavioural change component of the intervention was guided by Social Cognitive Theory which posits a role of environmental, individual, and behavioural factors on behaviour [40]. Environmental factors influencing eating habits will be addressed through the provision of recommended foods during the study period, prompts to consider individuals in the participants’ lives who could support them in their goals, and instruction to modify the physical environment. With respect to individual factors, we will increase knowledge about nutrition, food preparation, and the role of diet in mental health as well as increasing knowledge about participants’ own eating habits through self-monitoring. We will increase self-efficacy and confidence through motivational interviewing [41]. Regarding behaviour, participants will be provided with educational videos in order to improve their skills and ability to perform the desired behaviours (i.e., preparing nutritious food for themselves). We will also address habitual behaviours that are known to influence food choices in this population such as comfort eating.

### Educational material and resources

A set of educational handouts were developed for use in the EASe-GAD study (Table 1).

**Table 1:** EASe-GAD educational handouts

Handouts to be provided at the first diet counselling session	Handouts to be provided as indicated
-EASe-GAD Diet Recommendations and Rationale	-Caffeine and beverages
-Meal ideas	-Mindful eating
	-Managing cravings
	-Shopping list template
	-Tracking log

A collection of recipes was assembled as well as a collection of videos demonstrating recipe preparation, food preparation skills, and food safety videos. Participants will also receive shelf-stable food items at the first and fourth (in-person) visits in order to encourage adherence to the recommendations and decrease barriers to change.

### Dietary counselling

The dietary counselling sessions will be conducted by a licensed Naturopathic Doctor with more than 10 years of experience providing dietary counselling to individuals with mental health concerns and a certificate in motivational interviewing. The counselling sessions will aim to focus on the benefits of the recommended foods, highlighting the advantages of following the recommendations rather than the risks of following alternative diet patterns in order to maintain a positive message. The emphasis of the intervention will be on changes to dietary patterns; participants will be advised to consume the diet ad libitum with no restrictions on caloric intake.

In addition to providing education about nutrition, there will be significant efforts to support behaviour change (Supplemental file 3: Summary of Behaviour Change Techniques). There will be an emphasis on providing encouragement, communicating that diet changes are possible among individuals experiencing mental illness, and evoking past behaviour change successes. Goal setting will include a focus on setting measurable, specific, and realistic goals that are generated through a guided discussion with the participants and specific action planning related to the goal. Additionally, participants will be guided in identifying barriers to the achievement of the goal and strategies to overcome barriers. Participants will be instructed to modify their physical environment so that the encouraged foods are easy to access and require limited preparation and discouraged foods are less accessible. Because comfort eating is recognized as a driver of diet choices among the study population, participants will complete exercises related to mindful eating (ex. the raisin exercise) and review strategies for managing non-hunger cravings to eat such as grounding, acknowledging feelings, and engaging in enjoyable or distracting activities. The dietary counselling activities to be completed at each study visit are listed in Table 2.

**Table 2:** Dietary counselling activities to be completed at each study visit

Study visit	Dietary counselling activities
First dietary counselling session	-Inquiry about usual meals, beverages, aversions, preferences, cultural influences on eating
	-Presentation of EASe-GAD recommendations and rationales
	-Affirmation of aspects of diet that are already aligned with recommendations
	-Guided discussion where the participant identifies an opportunity for improvement without judgement
	-Creation of a goal, identification of possible barriers, and ways to navigate these barriers
Follow-up dietary counselling sessions	-Participant queried about the previous goal
	-If achieved, affirmations
	-If not achieved, discussion of obstacles or challenges, generation of problem-solving strategies while maintaining a positive message
	-Presentation of new material (educational handouts, resources) as appropriate
	-Creation of a new goal or adjustment of the previous goal
Final dietary counselling session	-Discussion about achievements made during the program and affirmations
	-Discussion of long-term goals and strategies to maintain

### Dietary supplement

During the 12-week study period, participants will be supplied with an omega-3 capsule supplement (AquaOmega High EPA) and instructed to take a dose of four capsules, once daily (containing a total of 3456 mg of omega-3 fatty acids: 2659 mg of Eicosapentaenoic acid (EPA), 532 mg of docosahexaenoic acid (DHA), and 800 IU of vitamin D), with a meal. Significantly more trials have been completed using omega-3 supplementation in the treatment of depressive disorders than anxiety disorders [42]. Recent guidelines recommend a daily dose of 1–2 g of EPA with a ratio of EPA:DHA of at least 2:1 for the treatment of depression [43]. Due to the lack of specific recommendations on the dose of omega-3 used for anxiety, our intervention will draw inference from these guidelines on depression to employ a dose that has been shown to be beneficial in other psychiatric disorders. We have selected a high dose, high EPA formula in order to maximize the likelihood of detecting a signal of effect in the present pilot trial.

### Concomitant therapies

All concomitant medication, psychotherapy, or natural health products will be allowed during this trial. In order to minimize the risk of adverse events related to hypervitaminosis D, participants who are supplementing 1701 to 4000 IU of vitamin D daily at the time of screening will be eligible to participate if they agree to decrease their vitamin D supplementation to 1700 so that their total daily intake of vitamin D, including the study product, is 2500 IU. Participants will be encouraged to continue all other previous treatments at the same dose for the duration of the trial, if possible, but allowed to make changes if recommended by their mental health care provider. Participants will be queried at baseline and each follow-up visit about their use of medication, psychotherapy, or natural health products, and any changes will be recorded, analysed, and reported.

### Safety and criteria for withdrawal

As rescue medicine, participants will be advised to take the over-the-counter product, diphenhydramine (Benadryl), in the event of an allergic reaction to the study product or any of the new foods that they consume, as recommended on the product label. Diphenhydramine will be provided to participants with the study product. Participants will be queried about the use of the rescue medication and asked to return any unused product at the end of the trial. The use of rescue medication for an allergic reaction will be recorded. Participants will be instructed to contact the study coordinator immediately if they experience a negative reaction to the study product or a serious worsening of their health. They will be provided with an email and phone number for this purpose. The qualified investigator will assess the patient and recommend a course of action. If the participant requires medical visits or medications that are not reimbursed by the Ontario Health Insurance Plan (OHIP), these costs will be reimbursed by the study sponsor, the Canadian College of Naturopathic Medicine. At each study visit, participants will be asked if they have experienced any negative effects since beginning the study product. Participants will be withdrawn from the study if they experience an allergic response, or other serious adverse event, as a result of the study product. These participants will be referred to their primary healthcare provider or a walk-in clinic for management. Participants will also be withdrawn if the trial psychiatrist deems the participant not adequately stable to continue to participate in the trial. This includes the presence of severe suicidal ideation or symptoms of psychosis. At each follow-up visit, participants will be asked by the intervention deliverer if they have experienced a significant worsening in their mental health since the previous visit. If they report symptoms related to suicidal ideation, the Ask Suicide-Screening Questionnaire (ASQ) will be completed [44]. If the participant screens positive, they will be referred to the study psychiatrist for a complete assessment. If they screen acutely positive, they will be referred directly to a crisis centre or the hospital emergency room. If they report any symptoms related to psychosis (any report of hallucinations, delusions, or disordered thinking), the participant will be referred to the study psychiatrist for assessment. If they meet the criteria for withdrawal, they will be referred to their current mental healthcare provider for appropriate assessment and treatment. If a participant reports a significant worsening in symptoms of anxiety or depression, they will be permitted to remain enrolled in the study. They will be encouraged to contact their primary mental health care provider for assessment and management. If the mental health care provider suggests that psychiatric rescue medication is needed, this clinician will recommend the treatment to the participant. If rescue medication is used for mental health symptoms, participants will be allowed to continue participating in the study. The use of rescue medication will be queried at each study visit and recorded. Given that the primary objective is feasibility and acceptability, participants with poor compliance with the study intervention will not be withdrawn from the study.

Expected adverse events for this study product include a fishy aftertaste, bad breath, heartburn, nausea, or loose stools, atrial fibrillation, and increased bleeding. All adverse events will be recorded, and these records will be maintained for 15 years. The clinical trial sponsor will inform the Natural and Non-prescription Health Product Directorate of Health Canada of any serious expected or unexpected adverse events related to the study product immediately if possible and no later than 7 days after becoming aware of the information. Serious adverse events will also be reported to the Research Ethics Board which has reviewed the study protocol.

Participants will be withdrawn from the study if their baseline serum levels of 25-hydroxy-vitamin-D are greater than 120 nmol/L, as this level has been associated with safety concerns [45]. These individuals will be instructed to contact their primary healthcare provider for assessment and management. In the event that the baseline or final blood work assessment suggests the presence of an undiagnosed medical condition, the participant will be notified immediately that there is an abnormal finding and told to contact their primary healthcare provider for appropriate assessment and treatment. With the participants’ permission, the lab reports will be sent to the healthcare provider. The participant will be allowed to continue to participate in the study.

### Outcomes measured

As the intervention is a pilot study, process evaluation (feasibility and acceptability) will be the primary outcome, helping to inform a future full-scale trial. The time to recruit 50 participants will be evaluated as part of the assessment of feasibility. In order to assess acceptability, the number of sessions attended will be counted. All participants will be asked to complete a brief satisfaction survey concerning the participant’s level of satisfaction with the program, the aspects which were helpful or unhelpful, and opportunities for improvement (Supplemental file 4: Outcome assessment). A sub-sample will be recruited to participate in a brief focus group in order to obtain more detailed qualitative responses about the participant experience and participant satisfaction. We will aim to recruit 8–18 participants to run 2–4 focus groups, each having 4–6 participants. After the second focus group, we will complete a brief data analysis and run additional focus groups if data saturation has not been achieved. The focus groups will be conducted using a semi-structured qualitative interview methodology. The sessions will be audio recorded and transcribed. Transcripts will be analysed using thematic analysis [46]. Additionally, we will assess the fidelity of the intervention by comparing the intervention protocol with the intervention delivery. The frequency of use of each questionnaire and resource will be counted. At baseline, a brief demographic questionnaire will capture information related to age, sex at birth, marital status, employment status, ethno-racial background, income, smoking status, and alcohol consumption (Supplemental file 4: Outcome assessment). Assessment of physical activity levels and food insecurity will also be completed using the International Physical Activity Questionnaire [47] and Canadian Community Health Survey Household Food Security Survey Module [48], respectively. Height and weight will be measured before and after the waitlist period and the intervention period. Body mass index and change in weight during the intervention and control period will be calculated. These data will characterize the study participants and allow for the assessment of confounding factors.

In order to assess changes in anxiety symptom severity, the Beck Anxiety Inventory, a validated, 21-item self-report inventory for measuring the severity of anxiety in participants with psychiatric illness, will be administered [49]. The impact on quality of life will be assessed using PROMIS-29 [50]. In order to assess the impact of the intervention on behaviour change and qualitatively assess the acceptability of the intervention, questionnaires related to mindful eating and diet quality will be administered before and after the intervention. Mindful eating behaviours will be measured using Mindful Eating Questionnaire (MEQ) which has been validated for use in a diverse adult population [51]. Changes in diet quality will be measured using the MEDI-LITE score, a concise tool for measuring consistency with the Mediterranean diet, which has been validated for use in adult populations [52]. Changes in self-efficacy will be assessed using the General Self-Efficacy Scale [53]. Changes in diet quality will also be assessed objectively through blood tests. Omega-3 fatty acids levels will be assessed using the dried blood spot test, Omega Score [54], which represents summed EPA, DHA, and Docosapentaenoic acid as a percentage of the total fatty acids in whole blood. The analysis will be completed by Lipid Analytical Laboratories. Additional assessment will be completed to measure fasting serum cholesterol panel, markers of blood sugar levels and regulation (Haemoglobin, fasting insulin, fasting glucose, and calculated HOMA-IR [55, 56]), vitamin C, beta-carotene, and C-reactive protein. These analyses will be completed by LifeLabs. Completion of the questionnaires and blood tests will be done at baseline, at the end of the waitlist control period (for participants assigned to this group) and at the end of the intervention. Participants who do not attend all of the scheduled visit but have not withdrawn will be contacted to complete the questionnaires and blood tests. See Supplemental file 4 for a summary of assessments on participant demographics, satisfaction with the dietary intervention, the research program, and participant experience.

Compliance with the supplement will be assessed at the final visit. Participants will be instructed to bring back all study product bottles containing any remaining capsules. The remaining capsules will be counted and recorded by a member of the study team. Compliance with the supplement will be reported as a percentage based on calculating the amount of pills consumed in comparison to the amount expected according to instructions.

### Statistical analysis

Since the pilot study is not powered to demonstrate efficacy, analysis of the pilot study results will be strictly descriptive. Baseline demographics and outcome measures will be summarized by group as means and confidence intervals, medians and interquartile ranges, or percentages and confidence intervals as appropriate. Process evaluation will be described both quantitatively (percent screened positive for inclusion, percent of eligible patients who consent to participate, percent with significant protocol violations) and qualitatively (e.g., reasons for failing inclusion criteria, reasons for refusing to participate, participant impressions of the intervention). All participants will be analysed in the group to which they were randomized. Missing data will be carefully documented to inform the design of the larger trial. Participants who complete the treatment but do not report a satisfaction level will be treated as ‘unsatisfied.’ No interim analysis will be undertaken. The study will be considered feasible if 50 participants are recruited within 2 years, and participants in the intervention group take at least 60% of the study product and attend at least 60% of the diet counselling sessions. The study will be considered acceptable if at least 70% of participants report that they were satisfied with the intervention. The statistical analysis plan for the full-scale trial is included in Supplemental file 5.

### Data management

The collection and storage of data will be done according to Good Clinical Practice Guidelines. All study staff involved in the collection or entry of data will be trained by the trial coordinator and will complete ethical conduct of research training (Tri-Council Policy Statement: Ethical Conduct of Research Involving Humans 2). Data from potential and enrolled participants will be stored in the secure platform, Research Electronic Data Capture (REDCap), and a password-protected database on a secure server managed by the Canadian College of Naturopathic Medicine. Data collected at each study visit will be captured using an electronic case report form which will be entered into the database. Questionnaire completion will be done by the study participants through REDCap. Laboratory data will be entered into the database and double-checked for accuracy by a team member blind to participant allocation. An audit of the data will be completed by a senior member of the research team who is not involved in data collection after 10 participants are enrolled or 3 months of time, whichever comes first. All information obtained during the trial will be kept in a locked cabinet or on a password-protected computer and destroyed (deleted or shredded) after 15 years.

### Access to data and dissemination

All personal health information will be kept confidential and only accessed by the research team, unless required by law. This includes an audit by the CCNM Research Ethics Board or Health Canada Natural and Non-prescription Health Products Directorate. All data will be maintained on password-protected, secure servers or locked filing cabinets for the required length of time. Following this time, data will be destroyed securely by deleting the digital files or shredding paper documents. No identifying information from any participant will be used in the dissemination of study results. Dissemination of the study findings will occur through publication. Study participants will have the option to be notified of the study findings.

## Discussion

Given the increasing attention to the importance of nutrition in mental well-being being communicated to the general public by the popular media, we expect that participants will be interested in the trial and that it will be feasible and acceptable. Given the abundance of observational data suggesting an important role of food choices in the development and progression of psychiatric disorders, in conjunction with the positive findings from recent intervention studies in depression, we expect that participation in the intervention will result in the improvement of anxiety symptoms.

At this present time, attention to the role of diet and lifestyle in guidelines for the prevention and treatment of psychiatric disorders is significantly lacking. This trial will build the foundation for larger, adequately powered studies which will add to the expanding evidence base attesting to the importance of nutrition in mental health care. This type of evidence creates a rationale for the inclusion of nutrition professionals in mental health care teams and the use of dietary counselling in the treatment of mental illness. It will open the door to shifting policy and increasing access. Nutrition interventions can be low in risk, acceptable to patients, cost-effective [10], and may have additional benefits to overall health. Nutritional counselling may be a way for nutrition professionals to contribute to meeting the needs of a patient population who report that presently available treatment options are often not accessible, effective, or tolerable, resulting in a decreased personal and social burden of GAD [4].

### Supplementary Information

**Additional file 1: Supplementary file 1.** SPIRIT 2013 Checklist: Recommended items to address in a clinical trial protocol and related documents*.

**Additional file 2: Supplemental file 2.** SPIRIT Figure: The schedule of enrolment, interventions, and assessments. **Supplemental file 3.** Summary of Behaviour Change techniques used and coding according to Behaviour Change Techniques (BCT) Taxonomy Version 1. **Supplemental file 4.** Outcome assessment. **Supplemental file 5.** Statistical analysis plan for the full-scale trial.

## Data Availability

The datasets used and/or analysed during the current study are available from the corresponding author on reasonable request.

## Abbreviations

GAD: Generalized anxiety disorder
EPA: Eicosapentaenoic acid
DHA: Docosahexaenoic acid
REDCap: Research Electronic Data Capture
EASe-GAD: Eating and Supplementation for Generalized Anxiety Disorder study
PROMIS: Patient Reported Outcomes Measurement Information System

## References

[1] Gale CK, Millichamp J. Generalised anxiety disorder. BMJ Clin Evid. 2007;2007. https://www.ncbi.nlm.nih.gov/pmc/articles/PMC3275153/. PMC2943796 19450347

[2] Baxter AJ, Scott KM, Vos T, Whiteford HA (2013). Global prevalence of anxiety disorders: a systematic review and meta-regression. Psychol Med.

[3] Saarni SI, Suvisaari J, Sintonen H, Pirkola S, Koskinen S, Aromaa A (2007). Impact of psychiatric disorders on health-related quality of life: general population survey. Br J Psychiatry.

[4] Collins KA, Westra HA, Dozois DJ, Burns DD (2004). Gaps in accessing treatment for anxiety and depression: challenges for the delivery of care. Clin Psychol Rev.

[5] Revicki DA, Travers K, Wyrwich KW, Svedsater H, Locklear J, Mattera MS (2012). Humanistic and economic burden of generalized anxiety disorder in North America and Europe. J Affect Disord.

[6] Vilela AA, Pinto Tde J, Rebelo F, Benaim C, Lepsch J, Dias-Silva CH (2015). Association of prepregnancy dietary patterns and anxiety symptoms from midpregnancy to early postpartum in a prospective cohort of Brazilian women. J Acad Nutr Diet.

[7] Fatemi F, Siassi F, Qorbani M, Sotoudeh G (2020). Higher dietary fat quality is associated with lower anxiety score in women: a cross-sectional study. Ann Gen Psychiatry.

[8] McMartin SE, Jacka FN, Colman I (2013). The association between fruit and vegetable consumption and mental health disorders: evidence from five waves of a national survey of Canadians. Prev Med.

[9] Francis HM, Stevenson RJ, Chambers JR, Gupta D, Newey B, Lim CK (2019). A brief diet intervention can reduce symptoms of depression in young adults - a randomised controlled trial. PLoS ONE.

[10] Jacka FN, O'Neil A, Opie R, Itsiopoulos C, Cotton S, Mohebbi M (2017). A randomised controlled trial of dietary improvement for adults with major depression (the 'SMILES' trial). BMC Med.

[11] Garcia-Toro M, Ibarra O, Gili M, Serrano MJ, Olivan B, Vicens E (2012). Four hygienic-dietary recommendations as add-on treatment in depression: a randomized-controlled trial. J Affect Disord.

[12] Bayes J, Schloss J, Sibbritt D. The effect of a Mediterranean diet on the symptoms of depression in young males (the “AMMEND: A Mediterranean Diet in MEN with Depression” study): A randomized controlled trial. Am J Clin Nutr. 2022;116(2):572–80. 10.1093/ajcn/nqac106 35441666

[13] Parletta N, Zarnowiecki D, Cho J, Wilson A, Bogomolova S, Villani A (2019). A Mediterranean-style dietary intervention supplemented with fish oil improves diet quality and mental health in people with depression: A randomized controlled trial (HELFIMED). Nutr Neurosci.

[14] Opie RS, O'Neil A, Itsiopoulos C, Jacka FN (2015). The impact of whole-of-diet interventions on depression and anxiety: a systematic review of randomised controlled trials. Public Health Nutr.

[15] Marx W, Moseley G, Berk M, Jacka F (2017). Nutritional psychiatry: the present state of the evidence. Proc Nutr Soc.

[16] Schwingshackl L, Schwedhelm C, Galbete C, Hoffmann G. Adherence to Mediterranean diet and risk of cancer: an updated systematic review and meta-analysis. Nutrients. 2017;9(10):1063. 10.3390/nu9101063 PMC5691680 28954418

[17] Rosato V, Temple NJ, La Vecchia C, Castellan G, Tavani A, Guercio V (2019). Mediterranean diet and cardiovascular disease: a systematic review and meta-analysis of observational studies. Eur J Nutr.

[18] Eleftheriou D, Benetou V, Trichopoulou A, La Vecchia C, Bamia C (2018). Mediterranean diet and its components in relation to all-cause mortality: meta-analysis. Br J Nutr.

[19] Su KP, Tseng PT, Lin PY, Okubo R, Chen TY, Chen YW (2018). Association of use of omega-3 polyunsaturated fatty acids with changes in severity of anxiety symptoms: a systematic review and meta-analysis. JAMA Netw Open.

[20] Egan AE, Seemiller LR, Packard AEB, Solomon MB, Ulrich-Lai YM (2019). Palatable food reduces anxiety-like behaviors and HPA axis responses to stress in female rats in an estrous-cycle specific manner. Horm Behav.

[21] Dallman MF, Pecoraro N, Akana SF, La Fleur SE, Gomez F, Houshyar H (2003). Chronic stress and obesity: a new view of "comfort food". Proc Natl Acad Sci U S A.

[22] Wardle J, Steptoe A, Oliver G, Lipsey Z (2000). Stress, dietary restraint and food intake. J Psychosom Res.

[23] Gibson EL (2012). The psychobiology of comfort eating: implications for neuropharmacological interventions. Behav Pharmacol.

[24] Penaforte FRO, Minelli MCS, Anastacio LR, Japur CC (2019). Anxiety symptoms and emotional eating are independently associated with sweet craving in young adults. Psychiatry Res.

[25] Finch LE, Tomiyama AJ (2015). Comfort eating, psychological stress, and depressive symptoms in young adult women. Appetite.

[26] Grunberg NE, Straub RO (1992). The role of gender and taste class in the effects of stress on eating. Health Psychol.

[27] Klein LC, Faraday MM, Quigley KS, Grunberg NE (2004). Gender differences in biobehavioral aftereffects of stress on eating, frustration, and cardiovascular responses 1. J Appl Soc Psychol.

[28] Nagase Y, Uchiyama M, Kaneita Y, Li L, Kaji T, Takahashi S (2009). Coping strategies and their correlates with depression in the Japanese general population. Psychiatry Res.

[29] Klump KL, Keel PK, Racine SE, Burt SA, Neale M, Sisk CL (2013). The interactive effects of estrogen and progesterone on changes in emotional eating across the menstrual cycle. J Abnorm Psychol.

[30] Nelson JB (2017). Mindful Eating: The Art of Presence While You Eat. Diabetes Spectr.

[31] Dalen J, Smith BW, Shelley BM, Sloan AL, Leahigh L, Begay D (2010). Pilot study: Mindful Eating and Living (MEAL): weight, eating behavior, and psychological outcomes associated with a mindfulness-based intervention for people with obesity. Complement Ther Med.

[32] Kinser PA, Robins JL (2013). Control group design: enhancing rigor in research of mind-body therapies for depression. Evid Based Complement Alternat Med.

[33] von Schacky C (2015). Omega-3 fatty acids in cardiovascular disease–an uphill battle. Prostaglandins Leukot Essent Fatty Acids.

[34] Sartorius N, Ustun TB, Lecrubier Y, Wittchen HU (1996). Depression comorbid with anxiety: results from the WHO study on psychological disorders in primary health care. Br J Psychiatry Suppl.

[35] Estruch R, Ros E, Salas-Salvado J, Covas MI, Corella D, Aros F (2013). Primary prevention of cardiovascular disease with a Mediterranean diet. N Engl J Med.

[36] Aucoin M, LaChance L, Naidoo U, Remy D, Shekdar T, Sayar N, Cardozo V, Rawana T, Chan I, Cooley K. Diet and anxiety: A scoping review. Nutrients. 2021;13(12):4418. 10.3390/nu13124418 PMC8706568 34959972

[37] Psaltopoulou T, Sergentanis TN, Panagiotakos DB, Sergentanis IN, Kosti R, Scarmeas N (2013). Mediterranean diet, stroke, cognitive impairment, and depression: a meta-analysis. Ann Neurol.

[38] Canada H. Eating well with Canada’s food guide 2019. Available from: http://www.hc-sc.gc.ca/fn-an/food-guide-aliment/index-eng.php. Accessed 1 Dec 2021.

[39] Opie RS, O'Neil A, Jacka FN, Pizzinga J, Itsiopoulos C (2018). A modified Mediterranean dietary intervention for adults with major depression: dietary protocol and feasibility data from the SMILES trial. Nutr Neurosci.

[40] Bandura A (1986). Social foundations of thought and action.

[41] Resnicow K, McMaster F (2012). Motivational Interviewing: moving from why to how with autonomy support. Int J Behav Nutr Phys Act.

[42] Liao Y, Xie B, Zhang H, He Q, Guo L, Subramanieapillai M (2019). Efficacy of omega-3 PUFAs in depression: A meta-analysis. Transl Psychiatry.

[43] Guu TW, Mischoulon D, Sarris J, Hibbeln J, McNamara RK, Hamazaki K (2019). International Society for Nutritional Psychiatry Research Practice Guidelines for Omega-3 Fatty Acids in the Treatment of Major Depressive Disorder. Psychother Psychosom.

[44] Horowitz LM, Bridge JA, Teach SJ, Ballard E, Klima J, Rosenstein DL (2012). Ask Suicide-Screening Questions (ASQ): a brief instrument for the pediatric emergency department. Arch Pediatr Adolesc Med.

[45] Ross AC, Manson JE, Abrams SA, Aloia JF, Brannon PM, Clinton SK (2011). The 2011 report on dietary reference intakes for calcium and vitamin D from the Institute of Medicine: what clinicians need to know. J Clin Endocrinol Metab.

[46] Braun V, Clarke V (2006). Using thematic analysis in psychology. Qual Res Psychol.

[47] Craig CL, Marshall AL, Sjostrom M, Bauman AE, Booth ML, Ainsworth BE (2003). International physical activity questionnaire: 12-country reliability and validity. Med Sci Sports Exerc.

[48] Bush M, General R. Canadian Community Health Survey, Cycle 2.2, Nutrition (2004): Income-Related Household Food Security in Canada. Health Canada, Ottawa, Canada. 2007.

[49] Leyfer OT, Ruberg JL, Woodruff-Borden J (2006). Examination of the utility of the Beck Anxiety Inventory and its factors as a screener for anxiety disorders. J Anxiety Disord.

[50] Hays RD, Spritzer KL, Schalet BD, Cella D (2018). PROMIS((R))-29 v2.0 profile physical and mental health summary scores. Qual Life Res..

[51] Framson C, Kristal AR, Schenk JM, Littman AJ, Zeliadt S, Benitez D (2009). Development and validation of the mindful eating questionnaire. J Am Diet Assoc.

[52] Sofi F, Dinu M, Pagliai G, Marcucci R, Casini A (2017). Validation of a literature-based adherence score to Mediterranean diet: the MEDI-LITE score. Int J Food Sci Nutr.

[53] Chen G, Gully SM, Eden D (2001). Validation of a new general self-efficacy scale. Organ Res Methods.

[54] Liu G, Mühlhäusler BS, Gibson RA (2014). A method for long term stabilisation of long chain polyunsaturated fatty acids in dried blood spots and its clinical application. Prostaglandins Leukot Essent Fatty Acids.

[55] Esteghamati A, Ashraf H, Khalilzadeh O, Zandieh A, Nakhjavani M, Rashidi A (2010). Optimal cut-off of homeostasis model assessment of insulin resistance (HOMA-IR) for the diagnosis of metabolic syndrome: third national surveillance of risk factors of non-communicable diseases in Iran (SuRFNCD-2007). Nutr Metab (Lond).

[56] Calkin CV, Ruzickova M, Uher R, Hajek T, Slaney CM, Garnham JS (2015). Insulin resistance and outcome in bipolar disorder. Br J Psychiatry.

