# Supplementary material for: Dietary counselling plus omega-3 supplementation in the treatment of generalized anxiety disorder: protocol for a randomized wait-list controlled pilot trial (the “EASe-GAD Trial”)
Source: Pilot Feasibility Stud. 2023 Nov 10;9:186. doi: 10.1186/s40814-023-01414-y (PMC10636887; doi:10.1186/s40814-023-01414-y)
Supplement: Supplementary file 2 — Additional file 2: Supplemental file 2. SPIRIT Figure: The schedule of enrolment, interventions, and assessments. Supplemental file 3. Summary of Behaviour Change techniques used and coding according to Behaviour Change Techniques (BCT) Taxonomy Version 1. Supplemental file 4. Outcome assessment. Supplemental file 5. Statistical analysis plan for the full-scale trial. [file 40814_2023_1414_MOESM2_ESM.docx]

**Supplemental File 2**

**SPIRIT Figure: The schedule of enrolment, interventions, and assessments.**

|  |  | **STUDY PERIOD** | | | | | | | | | |
| --- | --- | --- | --- | --- | --- | --- | --- | --- | --- | --- | --- |
|  | **Enrolment** | **Allocation** | **End of Wait List** | **Post-allocation** | | | | | | | **Close-out** |
| **TIMEPOINT (Days)** | ***Prior to allocation*** | **0** | **85** | ***1 or 86*** | ***15 or 100*** | ***29***  ***or 114*** | ***43 or 128*** | ***57 or 142*** | ***71 or 156*** | ***85 or 160*** | ***85+ or 160+*** |
| **ENROLMENT:** |  |  |  |  |  |  |  |  |  |  |  |
| **Eligibility screen** | X |  |  |  |  |  |  |  |  |  |  |
| **Structured Clinical Interview administered by a psychiatrist** | X |  |  |  |  |  |  |  |  |  |  |
| **Omega Score** | X |  |  |  |  |  |  |  |  |  |  |
| **Informed consent** | X |  |  |  |  |  |  |  |  |  |  |
| **Allocation** |  | X |  |  |  |  |  |  |  |  |  |
| **INTERVENTIONS:** |  |  |  |  |  |  |  |  |  |  |  |
| ***Dietary Counselling*** |  |  |  | X | X | X | X | X | X | X |  |
| ***Omega-3 Supplement*** |  |  |  |  |  |  |  |  |  |  |  |
| **ASSESSMENTS:** |  |  |  |  |  |  |  |  |  |  |  |
| ***Satisfaction Survey*** |  |  |  |  |  |  |  |  |  |  | X |
| ***Attendance*** |  |  |  | X | X | X | X | X | X | X |  |
| ***MEDI-LITE score*** |  | X | W |  |  |  |  |  |  |  | X |
| ***Beck Anxiety Inventory*** |  | X | W |  |  |  |  |  |  |  | X |
| ***Omega Score*** |  | X | W |  |  |  |  |  |  |  | X |
| ***Mindful Eating Questionnaire*** |  | X | W |  |  |  |  |  |  |  | X |
| ***PROMIS-29*** |  | X | W |  |  |  |  |  |  |  | X |
| ***International Physical Activity Questionnaire*** |  | X | W |  |  |  |  |  |  |  | X |
| ***Food Security Assessment*** |  | X |  |  |  |  |  |  |  |  |  |
| ***General Self-Efficacy Scale*** |  | X | W |  |  |  |  |  |  |  | X |
| ***Adverse Events*** |  |  |  | X | X | X | X | X | X | X | X |
| ***Height and Weight*** |  | X | W |  |  |  |  |  |  |  | X |
| ***Lipid panel, Hemoglobin A1C, fasting insulin, fasting glucose, vitamin C, beta-carotene, C-reactive protein*** |  | X | W |  |  |  |  |  |  |  | X |
| ***Compliance*** |  |  |  |  |  |  |  |  |  |  | X |

X: All participants W: participants allocated to the waitlist arm

Supplemental File 3: Summary of Behaviour Change techniques used and coding according to Behaviour Change Techniques (BCT) Taxonomy Version 1

| Grouping | Behaviour Change Technique | Intervention Component |
| --- | --- | --- |
| Goals and Planning | 1.1 Goal setting (behaviour) | Participants will set goals based on diet recommendations (ex. Eat fish twice per week) |
|  | 1.2 Problem solving | Discussion of barriers that may interfere and strategies to overcome the barriers |
|  | 1.4 Action Planning | Facilitator will guide participant in selecting meal ideas and recipes based on goal and set a plan to consume those meals on certain days of the week |
|  | 1.7 Review behaviour goal(s) | Follow up visit will involve a review of the previous week’s goals and based on the outcome, future goals will be generated or modified |
| Feedback and Monitoring | 2.3 Self-monitoring of behaviour | Participants will be given a form to record their intake of the recommended foods on a daily/weekly basis. This exercise will be optional. |
| Social Support | 3.1 Social support (unspecified) | Participants will be encouraged to identify a person in their life that could assist them in achieving the goal |
| Shaping Knowledge | 4.1 Instruction on how to perform a behaviour | Facilitator will explain cooking techniques or recipes that the participant is not familiar with. Provision of access to videos on food preparation skills |
|  | 4.2 Information about antecedents | Discussion of how emotions can lead to eating behaviours |
|  | 4.4 Behavioural Experiments | Ask participants to try soothing or distracting activities when experiencing non-hunger food cravings |
| Natural Consequences | 5.6 Information about emotional consequences | Facilitator will present evidence suggesting a relationship between better quality diet and better metal health |
| Comparison of behaviour | 6.1 Demonstration of the behaviour | Provision of access to videos demonstrating cooking skills and recipe preparation |
| Associations | 7.1 Prompts/cues | Instructions to place recommended food items in visible locations in their kitchen |
| Repetition and substitution | 8.4 Habit reversal | Instructions to prepare meals at home rather than purchasing take out |
| Comparison of outcomes | 9.2 Pros and cons | Encourage participant to consider the pros and cons of current dietary habits if there is ambivalence to change |
| Reward and Threat | 10.4 Social Reward | Congratulate participant on achieving goals |
| Regulation | 11.2 Reduce negative emotions | Teach self-regulation and relaxation techniques for use during non-hunger cravings |
|  | 11.3 Conserving mental resources | Advise to prepare food in bulk, pre-portion meals or snacks, pre-plan meals or grocery lists |
| Antecedents | 12.1 Restructuring the physical environment | Advise to keep food that is meant to be eaten less often in location that is inconvenient to access. |
| Self-belief | 15.1 Verbal persuasion about capability | Tell participant that they can successfully modify their diet despite barriers such as mental health symptoms |
|  | 15.3 Focus on past success | Encourage participant to describe occasions where they have been able to eat healthy foods |

Supplemental File 4: Outcome assessment

**Demographic Questionnaire**

Age: (enter age in years)

Sex assigned at birth:

- Female
- Male
- Other

Marital status:

- Single
- Married/common-law
- Divorced
- Widowed
- Prefer not to answer

Employment status

- Employed full-time
- Employed part-time
- Self-employed
- Student
- Unemployed
- Retired
- Prefer not to answer

Ethnic Background

- Black (African, Afro-Caribbean, African Canadian descent)
- East/Southeast Asian (Chinese, Korean, Japanese, Taiwanese, Filipino, Vietnamese, Cambodian, Thai, Indonesian descent)
- Indigenous (First Nations, Metis, Inuk/Inuit descent)
- Latino (Latin American, Hispanic descent)
- Middle Eastern (Arab, Persian, Afghan, Egyptian, Iranian, Lebanese, Turkish, Kurdish descent)
- South Asian (East Indian, Pakistani, Bangladeshi, Sri Lankan, Indo-Caribbean descent)
- White (European descent)
- Another race category
- Do not know
- Prefer not to answer

Education: Please indicate your highest level of education completed

- No certificate, diploma or degree
- Highschool diploma or equivalency
- Apprenticeship or trades certificate or diploma
- College or other non-university certificate or diploma
- University diploma or certificate below bachelor level
- University certificate, diploma or degree at bachelor level or above

Income: Please indicate the range that includes your total annual household income.

- $10,000 or less
- $10,001 to $20,000
- $20,001 to $30,000
- $30,001 to $40,000
- $40,001 to $50,000
- $50,001 to $60,000
- $60,001 to $70,000
- $70,001 to $80,000
- $80,001 to $90,000
- $90,001 to $100,000
- Greater than $100,000

Smoking status

- Have you smoked cigarettes in the past year? Yes (If yes, 2 questions below)
- No

On the days that you smoke, how soon after you wake up do you have your first cigarette?

1. Within 5 minutes
2. 6- 30 minutes
3. 31-60 minutes
4. After 60 minutes

How many cigarettes do you typically smoke per day?

1. 10 or fewer
2. 11-20
3. 21-30
4. 31 or more

Alcohol consumption

- Average number of alcoholic beverages per week: ______

**Satisfaction Survey**

Diet Satisfaction

|  | Strongly Disagree | Disagree | Neutral | Agree | Strongly Agree |
| --- | --- | --- | --- | --- | --- |
| I enjoyed the foods I ate during this program. |  |  |  |  |  |
| The recommendations were easy to follow |  |  |  |  |  |
| I felt hungry while following the program recommendations. |  |  |  |  |  |
| I missed some of the foods that I used to eat while following these recommendations. |  |  |  |  |  |
| I think I will be able to follow these eating recommendations long-term. |  |  |  |  |  |
| Eating according to the recommendations is affordable, compared to my usual eating habits. |  |  |  |  |  |
| I believe my overall health and well-being improved because of changing my eating habits. |  |  |  |  |  |
| When dining out, it was easy to find foods on the menu that fit with the recommendations. |  |  |  |  |  |
| The recipes provided to me were helpful. |  |  |  |  |  |
| The educational materials provided to me were helpful. |  |  |  |  |  |
| The amount of time I spent preparing foods was reasonable. |  |  |  |  |  |

Program Satisfaction

|  | Strongly Disagree | Disagree | Neutral | Agree | Strongly Agree |
| --- | --- | --- | --- | --- | --- |
| My interactions with the research staff were professional. |  |  |  |  |  |
| I was comfortable and had time to discuss my thoughts, questions and concerns. |  |  |  |  |  |
| I felt as if my treatment plan was individualized to my goals, personal beliefs, values and cultural background. |  |  |  |  |  |
| I felt that it was easy to schedule appointments. |  |  |  |  |  |
| E-mails and phone calls were responded to in a timely manner. |  |  |  |  |  |
| I would recommend a family member or friend to participate in this study. |  |  |  |  |  |
| I was satisfied with the Omega-3 supplement. |  |  |  |  |  |
| I would recommend the Omega-3 supplement to a friend or family member. |  |  |  |  |  |
| Overall, my experience during this study was positive. |  |  |  |  |  |

Open text questions:

1. What did you like most about participating in this study?
2. What did you not like, or like least, about participating in this study?
3. Which resources were most helpful? (examples: recipes, handouts, educational materials, videos etc.)
4. Which resources were least helpful?
5. Do you feel that your expectations were met during this study? Please explain.
6. Do you have any further comments related to the how the study was conducted? (ex. the processes, the questionnaires, etc.)
7. Were there any things that you liked about taking the Omega-3 supplement?
8. Were there any things that you did not like about taking the Omega-3 supplement?
9. How can we make this study better in the future?
10. Any other comments, concerns, or suggestions?

Supplemental File 5: Statistical analysis plan for the full-scale trial

For the full-scale trial, the primary outcome (Beck Anxiety Inventory) will be analyzed in a two-stage manner. All participants will be analyzed in the group to which they were randomized. First, the change in anxiety will be compared between the active group and the waitlisted controls at the completion of the 12-week intervention for the active group. Between-group differences will be tested using an ANCOVA analysis that compares 12-week anxiety measures between groups while controlling for baseline anxiety measure. The change in anxiety score from just prior to the 12-week intervention to just afterwards will be summarized for all patients, waitlisted or not, who do receive the dietary intervention. This will permit a more precise estimate (smaller confidence interval) for the magnitude of the benefit while preserving the rigor of the randomized controlled trial. As a secondary analysis, the ANCOVA analysis will be repeated adjusting for age, income, education, food insecurity, smoking, body mass index, alcohol consumption and physical activity level.
